# A Rapid, Fluorescence Switch-On Biosensor for Early Diagnosis of Sorghum Mosaic Virus

**DOI:** 10.3390/bios12111034

**Published:** 2022-11-17

**Authors:** Zhenlong Han, Congyuan Yang, Dan Xiao, Yinfu Lin, Ronghui Wen, Baoshan Chen, Xipu He

**Affiliations:** 1 School of Chemistry and Chemical Engineering, Guangxi University, Nanning 530004, China; 2 College of Life Science and Technology, Guangxi University, Nanning 530004, China; 3 College of Agriculture, Guangxi University, Nanning 530004, China; 4 Guangxi Key Laboratory of Sugarcane Biology, Guangxi University, Nanning 530004, China

**Keywords:** sorghum mosaic virus, quantum dots, nanobiosensor, immunocomplex

## Abstract

For the first time, a nanobiosensor was established for Sorghum mosaic virus (SrMV) detection. The biosensor consists of cadmium telluride quantum dots (CdTe QDs) conjugated to the specific antibody (Ab) against SrMV coat protein (CP) and carbon quantum dots (C QDs) labeled with SrMV coat protein. The formation of the fluorophore-quencher immunocomplex CdTe QDs-Ab+C QDs-CP led to a distinct decrease in the fluorescence intensity of CdTe QDs. Conversely, the emission intensity of CdTe QDs recovered upon the introduction of unlabeled CP. The developed biosensor showed a limit of detection of 44 nM in a linear range of 0.10–0.54 μM and exhibited the strongest fluorescence intensity (about 47,000 a.u.) at 552 nm. This strategy was applied to detect purified CP in plant sap successfully with a recovery rate between 93–103%. Moreover, the feasibility of the proposed method was further verified by the detection of field samples, and the results were consistent with an enzyme-linked immunosorbent assay (ELISA). Contrarily to ELISA, the proposed biosensor did not require excessive washing and incubation steps, thus the detection could be rapidly accomplished in a few minutes. The high sensitivity and short assay time of this designed biosensor demonstrated its potential application in situ and rapid detection. In addition, the fluorescence quenching of CdTe QDs was attributed to dynamic quenching according to the Stern-Volmer equation.

## 1. Introduction

Sorghum mosaic virus (SrMV) is widely distributed and responsible for the mosaic symptom on sugarcane all over the world [1]. The severe infection of SrMV leads to an inhibition in the photosynthesis of sugarcane, resulting in dwarfing and even death [2]. Currently, SrMV is becoming the primary pathogen of sugarcane mosaic disease (SMD) in China’s major sugarcane-growing region, which is recognized as the most destructive and prevalent viral disease of sugarcane in China [3,4]. According to previous studies, the morbidity of SrMV is 47.9%, leading to a 3–50% reduction in sugarcane yield and a decrease of 6–14% in sucrose content, equating to an annual loss of over US$30 million in China [5]. The removal of infected crops in the early stages of infection is critical for effective disease prevention. To meet this demand, analysis technology capable of detecting SrMV in low concentrations with a short analysis time is required. 

Conventional methods for plant pathogen detection primarily rely on enzyme-linked immunosorbent assays (ELISA) and polymerase chain reactions (PCR) [5,6]. Despite their high sensitivity and specificity, ELISA suffers from the drawbacks of complex washing steps and a long reaction time, while PCR requires high-quality samples, expensive equipment, and skilled personnel [7,8]. Thus, there is still a growing desire to develop a simple, rapid, and sensitive detection method for the diagnosis and prevention of SrMV.

In recent years, fluorescence-based nanosensors have received tremendous attention in biological detection and diagnosis [9]. As a class of novel fluorescent nanoparticles, quantum dots (QDs) have been widely investigated for the rapid and sensitive detection of various pathogens such as infectious bursal disease virus [10], avian leukosis virus [11], Peste des petits ruminants virus [12], Ectromelia virus [13], Hepatitis B virus [14], Dengue virus [15], etc. It is worth quoting that QDs are gradually becoming ideal donors in fluorescence analysis due to their captivating optical characteristics of high luminous intensity, large Stokes shift, wider excitation spectra, symmetry, tunable, and narrow emission spectrum [16].

On the other hand, organic dyes [17,18,19] and nanoparticles [20,21,22] have been frequently applied as quenchers in the reported fluorescence sensors, while applications of QDs are relatively rare [23,24,25]. QDs show several advantages over the above materials, including facile synthesis, high resistance to photo-bleaching, large absorption cross-section, which designate them as promising quenchers in fluorescence analysis. Thus, the attempt to employ QDs as quenchers is of significance. 

**Scheme 1:**
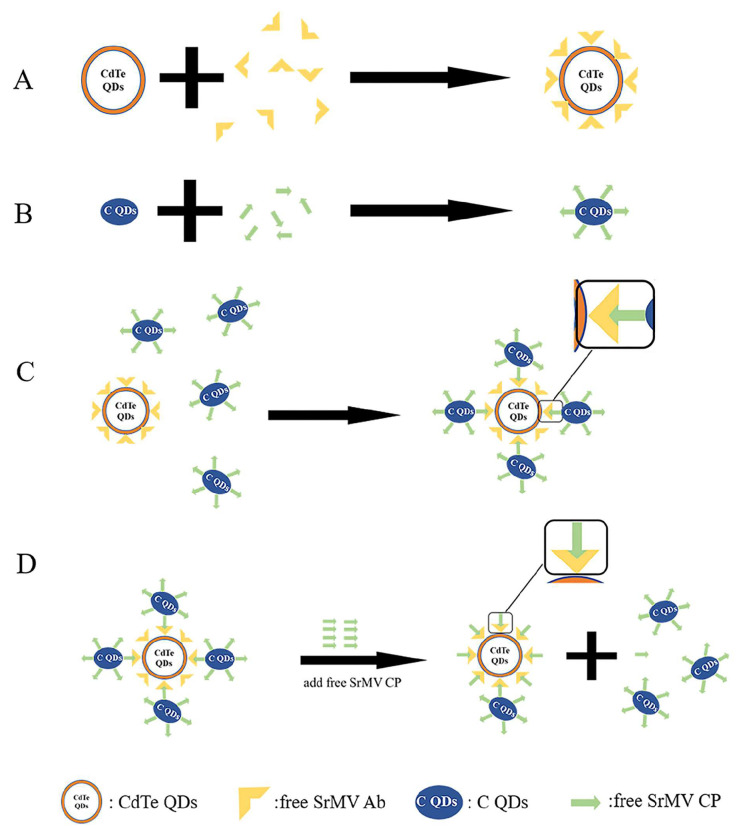
Assay mechanism of the developed biosensor.

In light of that, a QDs-based biosensor was developed for SrMV detection (Scheme 1). The biosensor was realized by the specific combination of carbon quantum dots (C QDs) conjugated to SrMV coat protein (CP) and CdTe QDs coupled with an anti-SrMV CP antibody (Ab). The formation of the immunocomplex CdTe QDs-Ab+C QDs-CP based on the mutual affinity between antibody and antigen gives rise to the attenuation in fluorescence intensity of CdTe QDs. At the detection stage, C QDs-CP was released from the antibody owing to the competitive replacement of free CP in the infected sample. Consequently, the fluorescence intensity of CdTe QDs recovered, and the resuming ratio was proportional to the concentration of the target. To the best of our knowledge, this is the first report on developing a nanobiosensor for SrMV detection.

## 2. Materials and Methods

### 2.1. Materials and Apparatus

Field samples were collected from different sugarcane varieties grown in the Fusui region, Guangxi province, China. Leaves from varying sugarcane plants were collected and stored at −20 °C until use. All chemicals, including cadmium chloride (CdCl_2_), mercaptoacetic acid (TGA), tellurium powder (Te), sodium borohydride (NaBH_4_), 1-(3-dimethyl aminopropyl)-3-ethyl carbodiimide hydrochloride (EDC), sodium citrate, urea, and thiourea, were purchased from Aladdin company (Shanghai, China). All used water was purified by an ultra-pure water purifier (Milli-Q plus 185, Millipore, Billerica, MA, USA).

All fluorometry studies were performed using a Tecan Infinite F200 micro-plate reader (Tecan, Mannedorf, Switzerland). All spectrophotometer analysis was performed using a Shimadzu UV-1800 Ultraviolet-visible spectroscopy spectrophotometer (Shimadzu, Kyoto, Japan). The transmission electron microscopy (TEM) images of the prepared QDs were acquired on a FEI Talos F200s (Hillsboro, OR, USA).

### 2.2. ELISA Procedure

The ELISA experiment was explored by using a slightly modified procedure [26]. Plant sap from infected sugarcane, healthy sugarcane, and a definite negative sample (as a negative control) was extracted by fully grinding 1 g of blades in liquid nitrogen followed by suspension in 500 μL of PBS buffer (pH = 7.4). The aforementioned extracts were added to the 96-well microtiter plate and stored at 4 °C overnight. Afterward, the plate was rinsed with PBST three times. Subsequently, the diluted Ab was coated on a 96-well microtiter plate, and the plate was incubated at 37 °C for 1 h and washed as described above. Following the addition of the diluted alkaline phosphatase-conjugated goat anti-rabbit immunoglobulin into each well, the plate was incubated at 37 °C for 1 h. Finally, 100 μL of a color-developing solution containing p-nitrophenyl phosphate (pNPP, 1 mg/mL) was added to the plate and incubated at room temperature for 30 min in the dark. The optical density at 405 nm was recorded for subsequent characterization. The experiments were repeated three times.

### 2.3. Preparation of TGA-Capped CdTe QDs

Hydrosoluble CdTe QDs were prepared based on previous typical literature [27]. NaHTe was prepared by reducing tellurium powder (0.1 g) with moderate NaBH_4_ (0.3 g) in 10 mL water under stirring and N_2_ bubbling. Subsequently, 0.4 g CdCl_2_·2.5 H_2_O and 250 μL TGA, as well as the freshly prepared oxygen-free NaHTe solution, were dissolved in 100 mL double-distilled water (pH adjusted to 10.0). The mixture was stirred and refluxed at 90 °C for 1 h under a nitrogen atmosphere. The crude product was deposited with ethanol and centrifuged at 8000 rpm for 10 min three times, and finally the pellet was lyophilized to obtain solid powder for further use.

### 2.4. Synthesis of Water-Soluble C QDs

C QDs were synthesized according to the reported literature with slight modification [28]. 0.25 g of Sodium citrate, 0.54 g urea, and 0.22 g thiourea were dissolved in 5 mL double-distilled water (at a molar ratio of 1:9:3). The mixture solution was placed in a stainless steel autoclave and heated at 180 °C for 6 h. After cooling down to room temperature, the yellow-brown carbon quantum dot solution was obtained and dialyzed with a dialysis tubing cellulose membrane (MWCO = 1000 Da) to remove the residual reactant.

### 2.5. Preparation of the CdTe QDs-Ab Conjugation

In order to conjugate CdTe QDs with Ab, CdTe QDs (20 mg/mL), Ab (0.3 mg/mL) and EDC (6.4 mg/mL) were mixed in 500 µL PBS buffer (pH = 7.4) and incubated at 28 °C in the dark for 2 h. The product was centrifuged at 12,000 rpm for 10 min to remove excess substances, and the supernatant was stored at 4 °C in the dark.

### 2.6. Preparation of The C QDs-CP Conjugation

400 µL prepared C QDs solution was first activated for 1 h in the presence of EDC (1.28 mg in 200 µL) and NHS (0.9 mg in 200 µL) at 37 °C in the dark. Subsequently, 50 μL of CP (1.8 mg/mL) was added drop by drop to the mixture and stirred gently at 4 °C for 2 h. Followed by the addition of Tris buffer (6 mg/mL, pH = 7.2) to terminate the reaction, the solution was centrifuged at 12,000 rpm for 5 min, and the upper phase was diluted to 500 μL and kept at 4 °C until use. A spectrophotometric analysis was performed to confirm the successful combination of C QDs and CP.

### 2.7. Determination of Quenching Efficiency

The quenching efficiency was calculated according to the Formula (1) [29], where F and F_0_ are the fluorophore emission intensities in the presence and absence of quenchers, respectively. To obtain the best quenching efficiency, a fixed amount of CdTe QDs-Ab and a serial dilution of the C QDs-CP were mixed, followed by the addition of PBS buffer to a final volume of 100 μL. That is to say, different C QDs-CP /CdTe QDs-Ab ratios were set for experiments at 1:25, 2:25, 3:25, 4:25, 1:5, 1.5:5, and 2:5. The experiments were repeated three times.

E = 1 − F/F_0_ (1)

### 2.8. Assessment of Nanobiosensors

To investigate the ability of the designed biosensor for SrMV detection, CdTe QDs- Ab and C QDs-CP in the optimum ratio were mixed in PBS buffer (pH = 7.4), and the solution was incubated at room temperature for 3 min to ensure the formation of the immunocomplex. The quenched emission intensity of the CdTe QDs was recorded at an excitation wavelength of 360 nm. Subsequently, different concentrations of purified CP were added to the mixture and incubated for 5 min. The subsequent fluorescence intensity was recorded in the same condition for CP quantification. To investigate the feasibility of the proposed method in the complicated biological environment, sap from healthy plants spiked with different concentrations of CP was examined. The experiments were repeated three times.

## 3. Results

### 3.1. Morphological and Spectral Characterizations of CdTe QDs and AuNP

**Figure 1:**
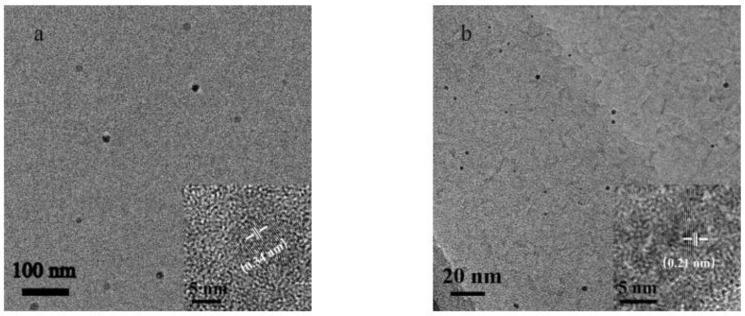
TEM images of CdTe QDs (**a**) and C QDs (**b**). Inset: High-resolution TEM images of corresponding nanomaterials.

The transmission electron microscopic (TEM) was used to characterize the morphology and size of QDs. As depicted in Figure 1a, the synthesized CdTe QDs were spherical and well dispersed, with an average diameter of 10 nm. The inset showed a lattice spacing distance of 0.34 nm, corresponding to the crystal face (111). Analogously, Figure 1b showed that the prepared C QDs were uniform and monodisperse with an average size of 5 nm. The inset revealed a lattice spacing distance of 0.21 nm attributed to the crystal face (100).

### 3.2. Structural Characterization of Quantum Dots

**Figure 2:**
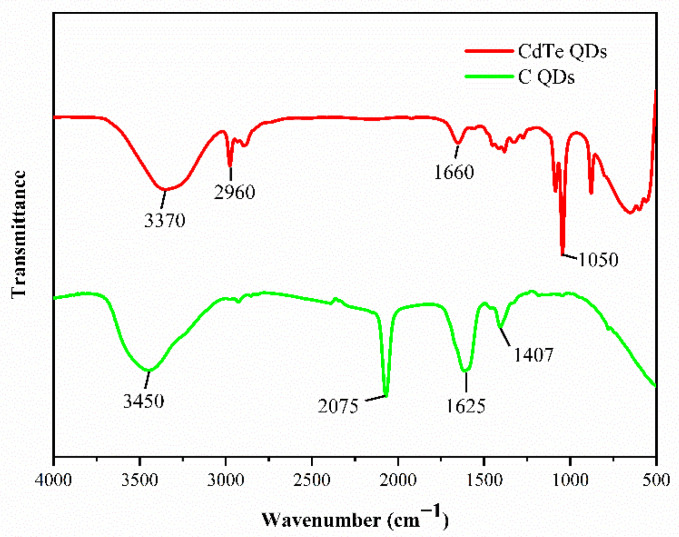
FT-IR spectra of CdTe QDs and C QDs.

A Fourier Transform Infrared Spectrometer (FT-IR) was carried out to characterize the functional groups on the surface of the obtained QDs. As shown in Figure 2, CdTe QDs exhibited the characteristic absorption bands related to O-H stretching vibration around 3370 cm^−1^ and C-H stretching vibration at 2960 cm^−1^, the bands at 1680 cm^−1^ and 1400 cm^−1^ were related to the stretching vibrations of C=O and C-H, respectively. Meanwhile, the absorption band appearing at 1050 cm^−1^ was related to the stretching vibration of C-S. The infrared spectrum of C QDs showed characteristic absorption peaks related to O-H and N-H stretching vibrations around 3450 cm^−1^, the band at 1625 cm^−1^ was attributed to the stretching vibration of C=O, while the bands at 1407 cm^−1^ and 2075 cm^−1^ were from the C-N and the N-H stretching vibrations, respectively. Taken together, these FT-IR data confirmed the presence of hydroxyl, carbonyl, or carboxyl groups on the surface of the QDs, which contributed to their water solubility [30].

### 3.3. Characterization of Bioconjugation

**Figure 3:**
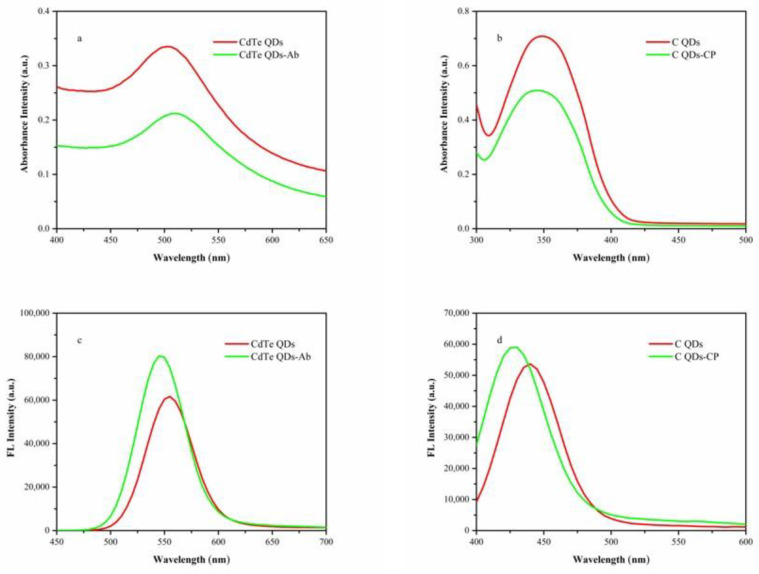
UV-Vis absorption spectra of CdTe QDs (**a**) and C QDs (**b**) before and after conjugation. Fluorescence spectra of CdTe QDs (**c**) and C QDs (**d**) before and after conjugation.

The bioconjugation of CdTe QDs with antibody and C QDs with antigen were confirmed by the following spectral analysis. The maximum absorption wavelengths of CdTe QDs were 501 nm and 509 nm before and after the introduction of the antibody, respectively. Meanwhile, the maximum OD values of CdTe QDs before and after the introduction of the antibody were 0.32 a.u. and 0.15 a.u., respectively. The bioconjugation of CdTe QDs with antibodies through covalent interactions was verified by the maximum peak shift and OD decrease of CdTe QDs-Ab. As to C QDs-CP, upon the introduction of antigen, a reduction in OD and variation on the maximum absorption wavelength (from 339 nm to 333 nm) confirmed the successful coupling of C QDs and CP (Figure 3b) [31]. Analogously, as shown in Figure 3c,d, the shift of the maximum emission wavelength and the increase of fluorescence intensity of QDs also confirmed the successful coupling [32]. In fact, amidation between the amino of protein and the carboxyl modified on the surface of QDs leads to an efficient coupling.

### 3.4. Biosensor Fabrication

**Figure 4:**
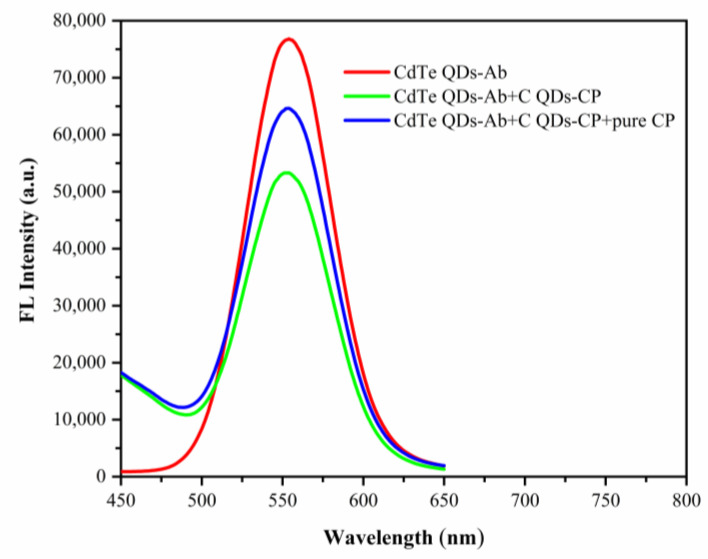
Fluorescence spectra of CdTe QDs-Ab, CdTe QDs-Ab+C QDs-CP, and CdTe QDs-Ab+C QDs-CP+pure CP.

As depicted in the sensing mechanism, based on the antibody-antigen interaction phenomenon, the fluorescence intensity of CdTe QDs-Ab decreased significantly owing to the formation of an immunocomplex. With the addition of pure CP, C QDs-CP was competitively replaced by unconjugated CP, which resulted in a marked increase in the fluorescence intensity of CdTe QDs-Ab (Figure 4). The proportion of fluorophore and quencher had a great effect on the accuracy and sensitivity of the system. Excessive CdTe QDs-Ab did not participate in the formation of the immunocomplex but reacted with the free CP in the sample, resulting in an underestimation of the target concentration. On the contrary, the existence of excessive C QDs-CP required higher amounts of free CP to overcome the competitive replacement in binding sites of the CdTe QDs-modified antibody, which may lead to a false negative result [31,33]. 

**Figure 5:**
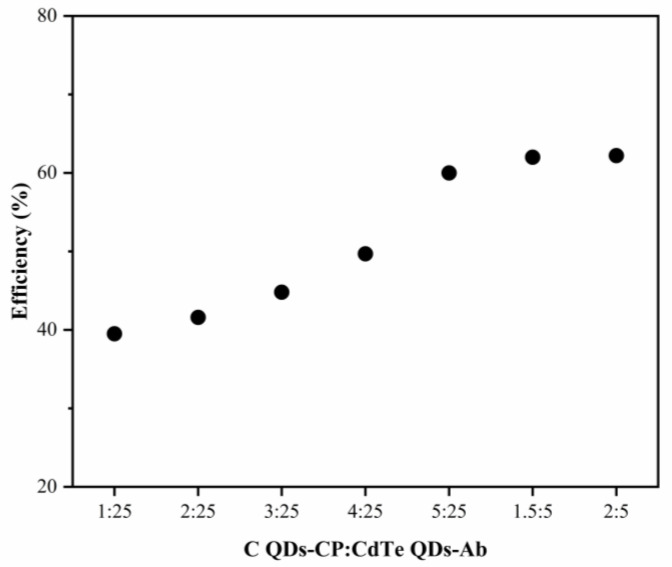
The effect of the proportion of CdTe QDs-Ab and C QDs-CP on the quenching efficiency of the developed biosensor.

The quenching efficiency of the designed system was investigated at different proportions of CdTe QDs-Ab and C QDs-CP. As shown in Figure 5, the optimum quenching efficiency was about 60% obtained at a C QDs-CP/CdTe QDs-Ab ratio of 1.5:5 where the fluorophore-quencher immunocomplex reached an quenching equilibrium. A ratio lower than optimum made the quenching efficiency decrease while a higher one kept the quenching efficiency constant.

### 3.5. Purified Sample Detection

**Figure 6:**
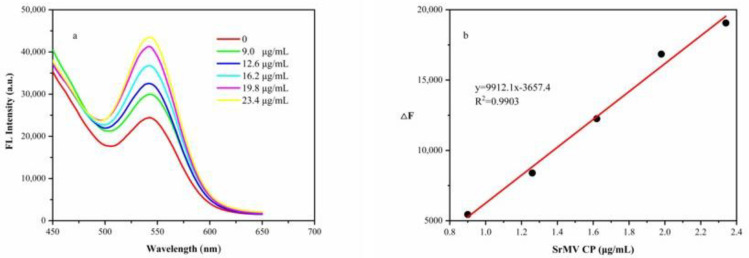
(**a**) The effect of CP concentrations on the fluorescent intensity of the developed biosensor and (**b**) Standard curve of the developed biosensor (∆F = F_t_ − F_0_).

As shown in Figure 6a, upon the addition of purified CP from 9 μg/mL to 23.4 μg/mL, the emission intensity of CdTe QDs increased gradually, accompanied by the increasing target concentrations. The corresponding linear equation was y = 9912.1x − 3657.4 with a correlation coefficient R^2^ of 0.9903 (Figure 6b). The limit of detection (LOD) was estimated at 2.2 μg/mL (LOD = 3S_0_/K, where S_0_ is the standard deviation of blank measurements (*n* = 3) and K is the slope of the calibration curve). The performance of other pathogenic factor biosensors was compared and presented as Table S1 in the Supplementary Materials.

The feasibility of this biosensor was investigated by detecting the known concentrations of CP in the plant sap (the extraction process was as described above). The result showed that the spiked recovery of CP was satisfactory (93–103%; Table 1).

### 3.6. Feasibility Verification

To investigate the ability of the proposed biosensor in field sample detection, 12 SrMV infected samples and 18 healthy samples were tested. Considering the extensive application of ELISA in plant virus detection, the performance of the designed biosensor was compared with ELISA. According to the ELISA procedure, samples with a measured absorption value(S)/negative absorption value (N) > 2.1 were judged positive, while those with S/N < 1.5 were judged negative. In addition, the sample with the aforementioned value between 1.5 and 2.1 was judged false negative. Results obtained by the ELISA method showed that 12 out of 12 positive samples were found positive and 18 out of 18 negative samples were found negative (Table 2). To facilitate comparison, we introduced a cut-off value as the criterion for a positive sample in the designed biosensor. The cut-off value was estimated at 18,842.506 by analyzing a negative control in three replications with X+3SD, where X is the average fluorescence intensity value of the negative sample and SD is the standard deviation. Samples with fluorescence value(s)/cut-off value (C) > 1 were judged as positive, while those with S/C values < 1 were judged negative. Results demonstrated that 12 out of 12 positive samples and 18 out of 18 negative samples were successfully detected by the proposed method, consistent with the results of the ELISA (Table 2).

### 3.7. Quenching Mechanism

To study the fluorescence quenching mechanism, the Stern–Volmer Equation (Formula (2)) was utilized to process the data [34].

**Figure 7:**
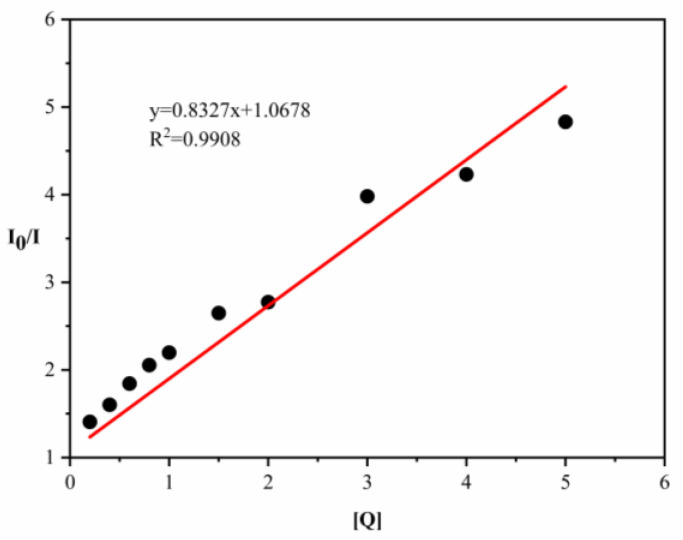
Stern-Volmer plots for the fluorescence quenching of CdTe QDs by C QDs.

I_0_/I = 1 + K_SV_[Q] = 1 + K_q_τ_0_[Q] (2)

where I_0_ and I are the fluorescence intensities of CdTe QDs in the absence and presence of quenchers, respectively. K_SV_ and K_q_ are the dynamic quenching constant and quenching rate constant of bimolecules, τ_0_ is the average lifetime of fluorescence molecules without quenching agents. Generally, K_q_ = K_SV_/τ_0_, and the τ_0_ of the CdTe QDs is taken as 2.6 × 10^−8^ s [35]. The Stern-Volmer quenching curve explained that the I_0_/I value of quantum dots was a function of the C QDs concentration [Q]. As shown in Figure 7, the Stern-Volmer plots were linear with a bimolecular quenching rate constant K_q_ value of 7.876 × 10^7^ L/(mol·s) which is less than 2.0 × 10^10^ L/(mol·s) [36]. This result indicated a dynamic type of quenching in this study [37].

## 4. Conclusions

As a golden standard technique, ELISA has been widely employed in detection procedures. However, this method has the limitation of time-consuming washing steps and complex operational processes. Herein, based on the specificity of the antibody-antigen and the optical characteristics of nanomaterials, we developed a novel nanobiosensor composed of CdTe QDs-Ab and C QDs-CP for SrMV detection. The proposed method enabled rapid diagnosis in complicated biological environments with a recovery rate between 93–103% and showed equivalent accuracy compared with ELISA. Moreover, the developed biosensor manifests several advantages over ELISA, including a short assay time and ease of operation. We believe that the assay reported here is a promising tool for large-scale screening procedures with great potential for the rapid detection of other plant viruses using corresponding antigens and relative antibodies.

## Figures and Tables

**Table 1:** Recoveries of CP from spiked samples.

Sample	Added (μg)	Measured (μg)	Recovery (%)	RSD (*n* = 3, %)
1	1.80	1.68 ± 0.22	93	13.35
2	2.88	2.70 ± 0.33	94	12.52
3	3.96	4.08 ± 0.41	103	10.26
4	5.04	4.88 ± 0.38	97	7.89
5	6.12	5.87 ± 0.51	96	8.72

**Table 2:** Detection results of field samples using the developed biosensor and an ELISA.

Sample	ELISA/ (O.D. Ratio)	Developed Sensor/ (a.u. Ratio)	Sample	ELISA/ (O.D. Ratio)	Developed Sensor/ (a.u. Ratio)
1	5.27	1.89	16	1.03	1.00
2	5.67	2.12	17	0.87	0.96
3	3.12	1.86	18	0.88	0.96
4	2.59	1.82	19	0.89	0.94
5	4.81	1.92	20	0.88	0.90
6	5.21	1.80	21	0.88	0.86
7	2.11	2.24	22	0.89	0.78
8	6.26	2.23	23	0.94	0.78
9	4.89	1.95	24	0.90	0.80
10	4.79	2.02	25	0.85	0.76
11	3.86	1.88	26	0.89	0.78
12	3.82	1.82	27	0.91	0.76
13	0.92	0.71	28	0.88	0.75
14	0.89	0.65	29	1.01	0.71
15	0.88	0.98	30	0.92	0.70

## Data Availability

Not applicable.

## Supplementary Materials

The following supporting information can be downloaded at: https://www.mdpi.com/article/10.3390/bios12111034/s1, Table S1: Comparison of the developed biosensor with previously reported methods for the pathogenic factor detection. Refs [10,27,31,33,38,39] are cited in Supplementary Materials.
