# Supplementary material for: A Rapid, Fluorescence Switch-On Biosensor for Early Diagnosis of Sorghum Mosaic Virus
Source: Biosensors (Basel). 2022 Nov 17;12(11):1034. doi: 10.3390/bios12111034 (PMC9688095; doi:10.3390/bios12111034)
Supplement: Supplementary file 1 [file biosensors-12-01034-s001.zip › biosensors-1993822-supplementary.pdf]

Supporting Information

A Rapid, Fluorescence Switch-On Biosensor for Early Diagnosis of Sorghum Mosaic Virus

Zhenlong Han [1], Congyuan Yang [1], Dan Xiao [2], Yinfu Lin [2], Ronghui Wen [2], Baoshan Chen [3,4] and Xipu He [1,4,*]

[1] School of Chemistry and Chemical Engineering, Guangxi University, Nanning 530004, China
[2] College of Life Science and Technology, Guangxi University, Nanning 530004, China
[3] College of Agriculture, Guangxi University, Nanning 530004, China
[4] Guangxi Key Laboratory of Sugarcane Biology, Guangxi University, Nanning 530004, China
* Correspondence: hexipu@gxu.edu.cn

Table S1. Comparison of the developed biosensor with previously reported methods for pathogenic factor detection.

Target	Linear Range (μM)	LOD (μM)	Correlation Coefficient	Reference
Citrus tristeza virus	Not reported	5.2×10^{-3}	0.98	[31]
Aflatoxin B1	$0.1 – 0.6 \times 10^{-3}$	2×10^{-5}	Not reported	[33]
Ganoderma boninense	Not reported	3.55×10^{-3}	0.935	[38]
Helicobacter pylori	0.01 – 0.2	4.5×10^{-3}	Not reported	[27]
Infectious bursal disease virus	0 – 0.6	3×10^{-2}	Not reported	[10]
Staphylococcus aureus	Not reported	1×10^{-3}	Not reported	[39]
Sorghum mosaic virus	0.10 – 0.54	4.4×10^{-2}	0.99	This work
